# Hydroxysafflor Yellow A Alleviates Ovalbumin-Induced Asthma in a Guinea Pig Model by Attenuateing the Expression of Inflammatory Cytokines and Signal Transduction

**DOI:** 10.3389/fphar.2019.00328

**Published:** 2019-04-05

**Authors:** Meng Zheng, Xinjing Guo, Ruiyan Pan, Jianwei Gao, Baoxia Zang, Ming Jin

**Affiliations:** Department of Pharmacology, Beijing Anzhen Hospital, Capital Medical University, Beijing Institute of Heart Lung and Blood Vessel Diseases, Beijing, China

**Keywords:** Hydroxysafflor yellow A, asthma, Th1, Th2, MAPK pathway, inflammation

## Abstract

Hydroxysafflor yellow A (HSYA) is an effective ingredient of the Chinese herb *Carthamus tinctorius* L. In this study, we aimed to evaluate the effects of HSYA on ovalbumin (OVA)-induced asthma in guinea pigs, and to elucidate the underlying mechanisms. We established a guinea pig asthma model by intraperitoneal injection and atomized administration OVA. Guinea pigs were injected intraperitoneally with HSYA (50, 75, 112.5 mg/kg) once daily from days 2 to 22 before OVA administration. We examined biomarkers including lung function, pulmonary histopathology, immunoglobulin E (IgE), Th1/Th2 relative inflammatory mediators, and related pathways. Pathological changes in lung tissues were detected by hematoxylin and eosin and periodic acid-Schiff staining. Phosphorylation levels of JNK mitogen-activated protein kinase (MAPK), p38 MAPK, ERK MAPK, and inhibitor of nuclear factor κBα (IκBα) were detected by western blot. plasma levels of total IgE, platelet-activating factor (PAF), and interleukin (IL)-3 were detected by enzyme-linked immunosorbent assay (ELISA). Expression levels of tumor necrosis factor (TNF)-α, IL-1β, IL-2, IL-4, IL-5, IL-6, IL-13, and interferon (IFN)-γ were detected by ELISA and real-time quantitative polymerase chain reaction. HSYA significantly reduced airway resistance, improved dynamic lung compliance, and attenuated the pathologic changes. HSYA also inhibited the phosphorylation of JNK MAPK, p38 MAPK, ERK MAPK, and IκBα, and inhibited the OVA-induced elevations of IgE, PAF, IL-1β, IL-6, IL-4, IL-5, and IL-13 and the decreases in TNF-α, IFN-γ, IL-2, and IL-3. These findings suggest that HSYA has a protective effect on OVA-induced asthma through inhibiting the Th1/Th2 cell imbalance and inhibiting activation of the MAPK signaling pathway.

## Introduction

Bronchial asthma is a chronic inflammatory airway disease characterized by airway inflammation, airway hyperresponsiveness (AHR), and airflow obstruction (Khorasanizadeh et al., 2017). More than 300 million people suffer from asthma worldwide (Gerez et al., 2010), with approximately 346,000 deaths annually (Lozano, 2012). The prevalence of asthma has recently increased dramatically, associated with environmental changes occurring throughout the world (Simpson et al., 2009; Swindle et al., 2009). However, the causes of asthma are multifactorial and complex, making it difficult to target therapeutically.

Numerous studies have identified an imbalance in Th1/Th2 responses as an important mechanism responsible for the induction and exacerbation of allergic asthma (Kidd, 2003; Wang J. et al., 2014; Huang C. et al., 2018). An excessive Th2 immune response has been observed in the pathogenesis of allergic asthma, with the overproduction of Th2-type cytokines, such as interleukin (IL)-4, IL-5, IL-6, and IL-13 (Zhou et al., 2015). Th2 cell-derived cytokines have also been implicated in immunoglobulin E (IgE) production, eosinophil migration and existence, mucus production, AHR, and tissue remodeling (Munitz et al., 2008; Lloyd and Hessel, 2010). In contrast, Th1-derived cytokines including interferon (IFN)-γ, IL-2, IL-3, and tumor necrosis factor (TNF)-α protected against allergic asthma (Liang et al., 2017). Evidence shows that the development of a healthy or allergic immune response is determined by the Th1/Th2 ratio, and immunoregulatory therapies that initiate a shift from Th2 to Th1 responses have been explored (Akdis et al., 2004; Thorburn and Hansbro, 2010). Glucocorticoids can effectively treat asthma by modulating the Th1 and Th2 responses (Hu et al., 2018), but their long-term use is associated with debilitating systemic side effects and potential exacerbation of Th2 immune responses (Stock et al., 2005). Overall, these observations provide strong evidence to suggest that regulating the imbalance in Th1/Th2 responses is an effective way to prevent the development and progression of asthma.

Mitogen-activated protein kinases (MAPKs) are ubiquitous and evolutionarily conserved enzymes known to act as a converging point for many upstream signaling pathways initiated by cytokine receptors, receptor tyrosine kinases, G protein-coupled receptors, and immunoreceptors such as T and B cell receptors (Khorasanizadeh et al., 2017). The MAPK family includes three different stress-activated protein kinase pathways: the p38 MAPK pathway, the c-Jun amino terminal kinase (JNK) MAPK pathway, and the extracellular regulated kinase (ERK) pathway (Davis, 1993). All three subfamilies of MAPKs have been implicated in the pathogenesis of asthma. p38 MAPK is the most extensively studied MAPK in asthma, and activation of p38 MAPK has been shown to cause eosinophil differentiation and activation (Adachi et al., 2000), mast cell migration (Ishizuka et al., 2001), IgE synthesis (Marshall et al., 1998), Th2 reaction aggravation (Huang L. et al., 2018), and airway remolding (Zhai et al., 2004). JNKs have also been implicated in the pathogenesis of asthma (Liang et al., 2016), and several pro-inflammatory genes, including adhesion molecules (Korenaga et al., 1997), growth factors (Hata et al., 2000), and intercellular adhesion molecule 1 (Lee et al., 2015) are regulated by the JNK MAPK pathway. Various *in vitro* studies have demonstrated the role of ERK MAPK in important aspects of asthma pathogenesis, including goblet cell hyperplasia (Atherton et al., 2003), eosinophil migration (Boehme et al., 1999), extracellular matrix protein secretion (Kazi et al., 2004), Th2 cytokine production (Puig-Kroger et al., 2001), and IgE production (Basaki et al., 2002). Furthermore, inhibiting the MAPK signaling pathway in pulmonary inflammatory cells (such as mast cells) was reported to have therapeutic potential in the treatment of allergic diseases such as asthma. Based on these results, we studied the anti-asthma effect of hydroxysafflor yellow A (HSYA) and discussed the involvement of MAPK pathways in its actions.

*Carthamus tinctorius* L. (safflower) is a traditional Chinese medicine that has been used to treat blood stasis for 1000s of years. HSYA is the main active ingredient in safflower, and has recently been shown to possess many pharmacological properties, including anti-inflammatory (Song et al., 2013; Jin et al., 2016a), antitumor (Sun et al., 2012), and antioxidant (Yang et al., 2015) effects, as well as exerting protective effects on the heart, brain, and nerves (Sun et al., 2012). We previously showed that HSYA attenuated the decreased oxygen saturation, edema, congestion, and expression of inflammatory factors associated with lipopolysaccharide-induced acute lung inflammation in mice (Zhang et al., 2017). Safflor Yellow injection, which contains >90% HSYA, was shown to alleviate bleomycin-induced lung fibrosis in rats (Wang et al., 2011). HSYA also inhibited transforming growth factor-β1-induced activation of ERK/MAPK signaling in MRC-5 cells (Pan et al., 2016). However, the effects of HSYA on asthma remain unclear. This study focused on the effects of HSYA on the Th1/Th2 balance and MAPK pathways in a guinea pig model of asthma. The results suggest that HSYA may represent a promising therapeutic strategy for asthma.

## Materials and Methods

### HSYA Preparation

**FIGURE 1:**
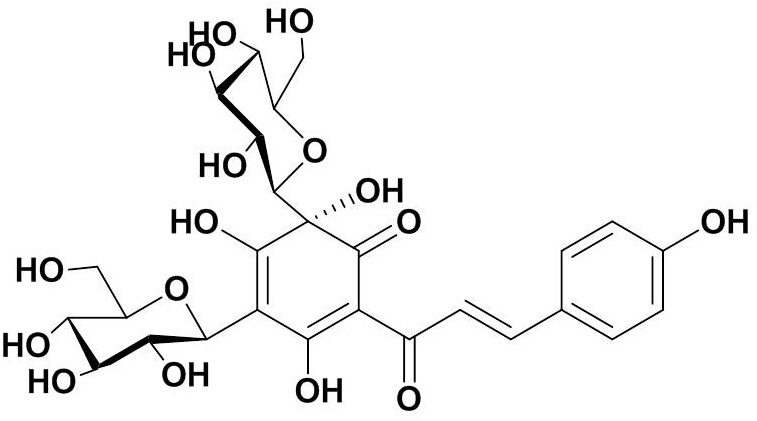
Molecular structure of Hydroxysafflor yellow A (HSYA).

Safflower was purchased from Huahui Kaide Pharmaceutical, Co., Ltd. (Shanxi, China) and produced in Tacheng City, the Xinjiang Uygur Autonomous Region, China. It was identified by Professor Hongzhu Guo (Beijing Institute for the Control for Drug Control). HSYA was isolated from an aqueous solution of safflower extract using macroporous resin-gel chromatography, as described previously (Zang et al., 2008). The molecular weight of HSYA is 612 and the molecular structure is shown in Figure 1. HSYA was dissolved in aseptic normal saline for subsequent use.

**FIGURE 2:**
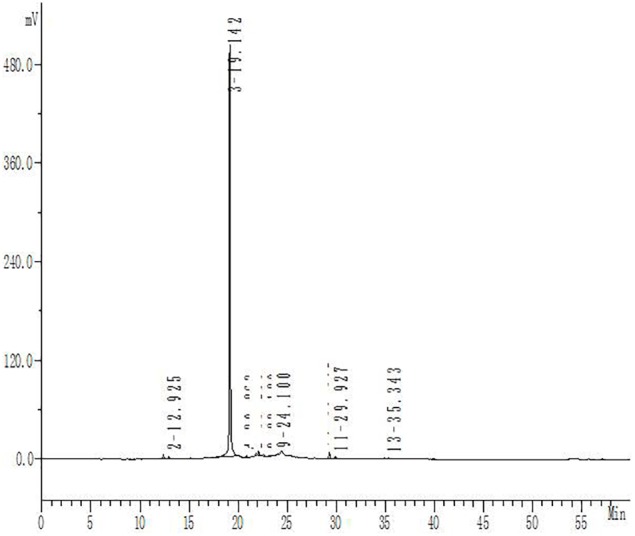
High-performance liquid chromatography analysis of HSYA. The absorbance was measured at 405 nm.

Hydroxysafflor yellow A purity was determined by high-performance liquid chromatography using an Apollo C18 reversed-phase column (250 mm × 4.6 mm, 5 μm; Grace Davison, Columbia, MD, United States) with a mobile phase of acetonitrile (A) and 0.1% trifluoroacetic acid (B), at a flow rate of 1 ml/min. The gradient elution program was as follows: solvent A was increased linearly from 1 to 35% from 0 to 50 min, and solvent B was decreased linearly from 99 to 65% over the same period; solvent A was then increased linearly from 35 to 45% from 50 to 60 min, while solvent B was decreased linearly from 65 to 55%. The optical absorbance was monitored at 405 nm and the column temperature was 30°C. The purity of HSYA for this study was 93% (Figure 2).

### Experimental Reagents

Ovalbumin (OVA) and methacholine (MCH) (Sigma-Aldrich, St. Louis, MO, United States) were dissolved in 0.9% NaCl. Dexamethasone (DXM) was produced by Tianjin Pharmaceutical, Co., Ltd. (Tianjin, China). TRIzol reagent and Moloney murine leukemia virus reverse transcriptase were purchased from Invitrogen (Carlsbad, CA, United States). The SYBR Premix ExTaq^TM^ (Perfect Real Time) kit was produced by Agilent Technologies (Santa Clara, CA, United States). Enzyme-linked immunosorbent assay (ELISA) kits were from Westang (Shanghai, China). p38 MAPK, phospho-p38 MAPK (Thr180/Tyr182), JNK MAPK, phospho-SAPK/JNK (Thr183/Tyr185), inhibitor of nuclear factor κBα (IκBα), and phospho-IκBα antibodies were from Cell Signaling Technology (Danvers, MA, United States). p44/42 MAPK (ERK1/2) and phospho-p44/42 MAPK (ERK1/2) (Thr202/Tyr204) antibodies were from YTHX Biotechnology (Beijing, China). GAPDH antibody was from Sigma-Aldrich.

### Animals and Drug Administration

Specific-pathogen-free mature male Hartley guinea pigs weighing 200–250 g were obtained from the Centre of Experiment Animals of China Institute for Food and Drug Control (Beijing, China) (Certificate of Conformity: Beijing Experimental Animal Testing by certificate: 11400500018342). Animals were maintained in the animal department of AnZhen Hospital under controlled temperature (23 ± 2°C) and humidity (60 ± 10%), with a 12 h light/dark cycle. All experimental procedures conformed to the Beijing Laboratory Animal Management Ordinance and were approved by the Committee on the Ethics of Animal Experiments of Capital Medical University.

A total of 140 male Hartley guinea pigs were used throughout the study, including 70 for the measurement of lung function and 70 for the detection of other indicators. The guinea pigs were all adapted for 1 week prior to the experiments, and were then divided randomly into seven groups according to body weight (guinea pigs weighing 200–250 g were divided into five levels with 10 g increments, each of the seven groups included guinea pigs from all of the five weight categories) (*n* = 10 each): normal control group; HSYA blank group (HSYA 112.5 mg/kg); OVA group; OVA+HSYA groups (HSYA 50, 75, 112.5 mg/kg); and OVA+DXM group (DXM 3 mg/kg). Sensitization of animals to OVA was performed according to previous study (Pejman et al., 2014). The guinea pigs in the OVA, OVA+HSYA (50, 75, 112.5 mg/kg), and OVA+DXM groups were sensitized by intraperitoneal (i.p.) injection of 100 mg OVA dissolved in saline on the 1st day and a further 10 mg on the 8th day. From day 16, sensitized animals were exposed to an aerosol of 1% OVA for 2 min daily for 7 days. Normal control animals and the HSYA blank group were treated similarly using saline instead of OVA. The aerosol was administered in a closed chamber (80 cm × 60 cm × 50 cm). From day 2 to day 22, animals in the OVA+HSYA and OVA+DXM groups were injected with the indicated doses of HSYA or DXM, respectively. Guinea pigs in the normal control group received an equal volume of normal saline and animals in the HSYA blank group received i.p. HSYA (112.5 mg/kg). The animals were sacrificed on day 23 after anesthetization with pentobarbital sodium, and samples were taken for subsequent analyses.

### Measurement of Lung Function

Airway hyperresponsiveness, as a key functional indicator for asthma, was measured using the AniRes 2005 lung function system (Bestlab, AniRes 2005, version 2.0, China) at 24 h after the final OVA challenge. The guinea pigs were weighed and i.p. pentobarbital sodium was administered at a dose of 75 mg/kg. When the animals showed no response to an acupuncture pin prick on their feet, the neck was incised to expose the trachea and jugular vein. A tracheal cannula was inserted and fixed using a suture. The guinea pig was then placed in the body plethysmograph chamber and the tracheal cannula was connected to the ventilator with an expiration/inspiration ratio of 20:10 and respiratory rate of 70 breaths/min. An injector needle was inserted into the jugular vein to administer five does of MCH (0, 0.025, 0. 05, 0. 1, 0.2 mg/kg), sequentially at 10-min intervals. Airway responsiveness was assessed using the indexes of expiratory resistance (Re), inspiratory resistance (Ri), and the minimum dynamic lung compliance (Cdyn). The baseline respiratory curve was recorded before MCH administration and the airway resistance and dynamic lung compliance curves were recorded after each MCH injection. The area between the recording curve and the baseline over 500 s was defined as Re or Ri, respectively.

### Histological Examination

A portion of left lung tissue was fixed in 10% formalin and stained with hematoxylin and eosin (HE). The rest of the lung was placed in Cary fixative for periodic acid-Schiff (PAS) staining. The samples were then sectioned at 4 μm and stained with HE or PAS according to conventional methods. Images were evaluated under a light microscopeand histological analyses were performed blindly.

### Western Blot Analysis

Frozen lung tissue (30 mg) was minced and homogenized in ice-cold lysis buffer containing Phosphatase Inhibitor Cocktail 3 (Sigma-Aldrich) and phenylmethanesulfonyl fluoride. The concentration of total protein was measured using a bicinchoninic acid assay kit according to the manufacturer’s instructions, with 30 μl protein per lane for separation by 10% sodium dodecyl sulfate-polyacrylamide gel electrophoresis, followed by transfer to a polyvinylidene difluoride membrane. The membrane was blocked with 5% non-fat dried milk and then incubated with p38 MAPK, phospho-p38 MAPK, JNK MAPK, phospho-JNK MAPK, ERK MAPK, phospho-ERK MAPK, IκB, phospho-IκB, and GAPDH antibodies, respectively (1:1000 dilution) at 4°C overnight. After washing three times with TBST, IRDye-conjugated goat anti-mouse or goat anti-rabbit antibodies (1:5000 dilution, LI-COR Biosciences, Lincoln, NE, United States) were added at room temperature for 1 h. The intensities of the bands were then scanned and visualized using an Odyssey infrared imaging system (Gene Company, Beijing, China).

### Real-Time Quantitative Polymerase Chain Reaction (RT-qPCR)

Total RNA was isolated from the lung tissues using TRIzol reagent (TRIzol), according to the manufacturer’s protocol. The concentration and quality of RNA were detected using a NanoDrop 2000 device (Thermo Scientific, Wilmington, DE, United States). RNA samples were then reverse transcribed into cDNA using a reverse transcriptase kit (Promega, Madison, WI, United States) with 2 μg RNA. The mRNA level of the target gene was quantified by RT-qPCR using a SYBR^®^ Premix ExTaq^TM^ kit on a Bio-Rad iCycler iQ5 Real-time Detection System (Bio-Rad, Hercules, CA, United States). The PCR process was as follows: initial denaturation at 95°C for 10 min, followed by 39 cycles of 95°C for 10 s, 55°C for 30 s, and finally 55 to 95°C in increments of 0.5°C every 10 s for melting curve analysis. The sense and antisense primer sequences are given in Table 1.

**Table 1:** Primers used for real-time quantitative PCR (RT-qPCR).

Name	Primer sequence
*Gapdh*	F: CCGGCCAAATACGATGACAT R: TGTAGCCCAAGATGCCTTTGAG
*IL6*	F: CTGAGCCCAACTCCAGATGC R: GGCGCTGAAGGACGATTTCA
*IL1b*	F: CATGAGCTTCGTACAAGGAGAAAG R: CAGGTACAGATTCTTCCCCTTGA
*Tnfa*	F: GGCCTACAGCTTTGATCCCTG R: AAAGGCTCCCTGGTCTCCAG
*Ifng*	F: ATGGGACGCTCTTTGTAGGC R: ACCTTGTTGCTGCTGTTGTCT
*IL2*	F: CTTGCACTTCTCACGAGCAGT R: AGTCGGTCCTGTGTTTGCTTT
*IL4*	F: CCAGCGTCCTGAGACAAGTG R: TCTTTCAGTGTCGTCTGCCG
*IL5*	F: GTTCCTGGATTACCTGCAAGAA R: GTCTCAGCCTTCAATTGTCCAT
*IL13*	F: TCCAACTGCAGCGCCC R: GGCCTTGTGCTGGCAAAG


Relative mRNA levels were calculated using the 2^-ΔΔCt^ method with data normalized to the GAPDH housekeeping gene.

### Enzyme-Linked Immunosorbent Assay

Blood was taken from the heart using a procoagulant tube, placed on ice, and then centrifuged at 1760 *g* for 20 min. Plasma levels of TNF-α, IL-1β, IL-2, IL-4, IL-5, IL-6, IL-13, IgE, platelet-activating factor (PAF), IL-3, and IFN-γ were measured by ELISA, according to the manufacturer’s protocols. The absorbance was measured at 450 nm using an ELISA reader (Epoch, Biotek, Winooski, VT, United States).

### Statistical Analysis

Data were analyzed using SPSS 19.0 software (SPSS, Inc., Armonk, NY, United States) by one-way analysis of variance (ANOVA) with Student–Newman–Keuls multiple comparison tests. Figures were generated using GraphPad Prism 5.0 (GraphPad Software, San Diego, CA, United States). All data were expressed as mean ± standard deviation (SD), and a *p*-value of < 0.05 was considered statistically significant.

## Results

### Effect of HSYA on Lung Morphology

**FIGURE 3:**
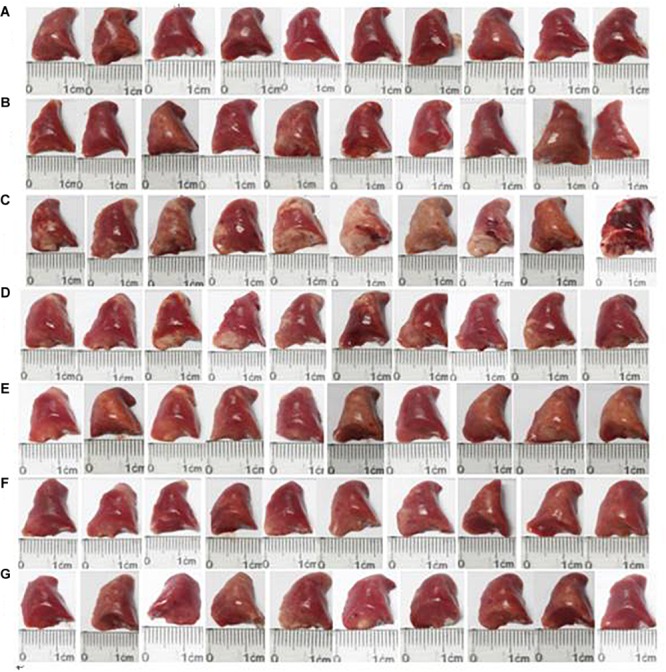
Effect of HSYA on lung morphology in guinea pigs. **(A)** Normal control; **(B)** HSYA blank; **(C)** OVA; **(D)** OVA+50 mg/kg HSYA; **(E)** OVA+75 mg/kg HSYA; **(F)** OVA+112.5 mg/kg HSYA; **(G)** OVA+3 mg/kg DXM.

We observed changes in the surface of the lungs (Figure 3). There was no hyperemia or edema in the lungs in the normal control group, and the lung texture was soft and uniform. However, hard white patches were evident on the lung in the OVA group, and the surface was rough, with congestion and swelling. HSYA alleviated these asthma-related changes in lung morphology in a dose-dependent manner, with high-dose HSYA (112.5 mg/kg) and DXM being particularly effective.

### Effect of HSYA on Lung Function

**FIGURE 4:**
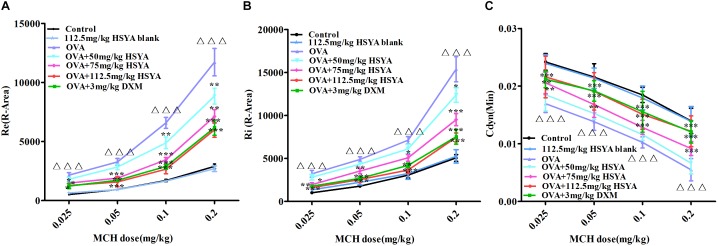
Effects of HSYA on OVA-induced AHR in guinea pigs. Effects of different doses of MCH on **(A)** relative area of Re; **(B)** relative area of Ri; **(C)** minimum value of Cdyn. Data presented as mean ± SD. ^∆∆∆^ *p* < 0.001 versus normal control group, ^∗^*p* < 0.05, ^∗∗^*p* < 0.01, ^∗∗∗^*p* < 0.01 versus OVA group.

Re and Ri increased in asthmatic guinea pigs while lung compliance decreased with increasing MCH concentration (Figure 4). Compared with the normal control group, guinea pigs in the OVA group showed a more pronounced airway response to MCH (*p* < 0.01). HSYA and DXM significantly reduced airway resistance and improved lung compliance in asthmatic guinea pigs (*p* < 0.01), except for the OVA+50 mg/kg HSYA group. There was no significant difference between the normal control and HSYA blank groups.

### Effect of HSYA on Lung Histopathology

**FIGURE 5:**
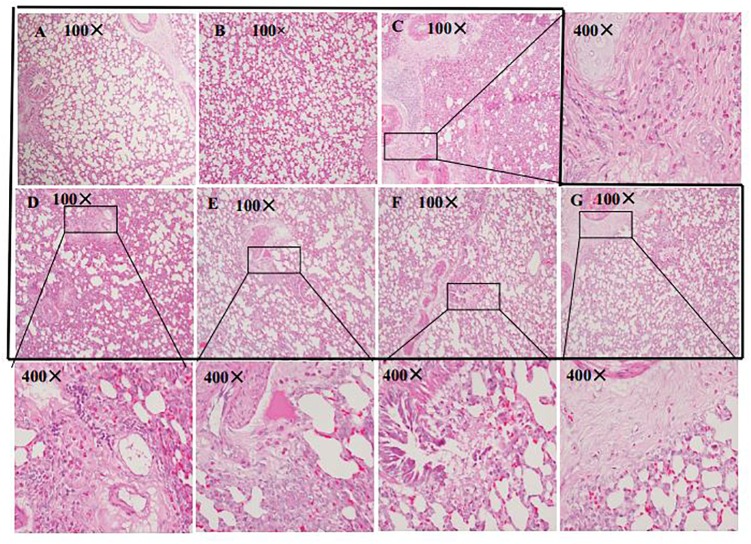
Histological changes in left lung tissue in guinea pigs by HE staining (original magnifications 100 and 400×). **(A)** Normal control; **(B)** HSYA blank; **(C)** OVA; **(D)** OVA+50 mg/kg HSYA; **(E)** OVA+75 mg/kg HSYA; **(F)** OVA+112.5 mg/kg HSYA; **(G)** OVA+3 mg/kg DXM.

Lung tissues remained intact in the normal control (Figure 5A) and HSYA blank groups (Figure 5B), and the alveolar space was clearly visible with no obvious inflammatory cell infiltration. In contrast, massive infiltration of inflammatory cells, alveolar collapse, and significant thickening of the interstitium were observed in the OVA group (Figure 5C). These histopathological changes were alleviated by HSYA in a dose-dependent manner, with no significant improvement following low-dose (50 mg/kg) HSYA (Figure 5D), but significant improvement following administration of 75 (Figure 5E) or 112.5 mg/kg HSYA (Figure 5F) or DXM (Figure 5G).

### Effect of HSYA on Mucus Secretion by Lung Tissue

**FIGURE 6:**
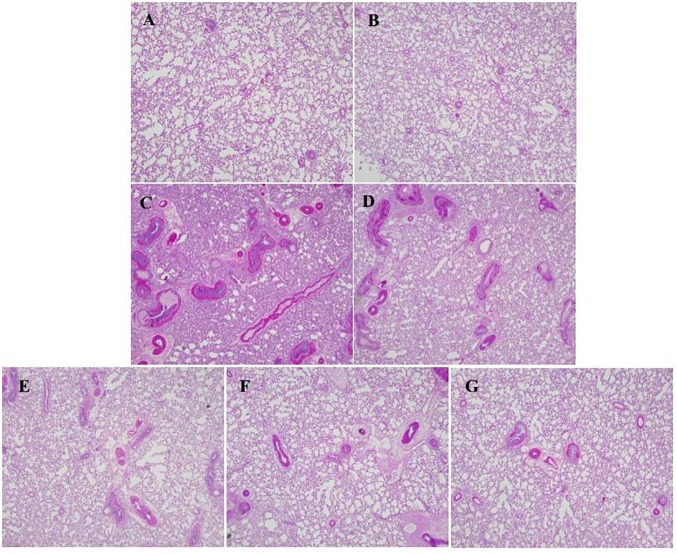
Effect of HSYA on mucus secretion in lung tissue by PAS staining (original magnification 100×). **(A)** Normal control; **(B)** HSYA blank; **(C)** OVA; **(D)** OVA+50 mg/kg HSYA; **(E)** OVA+75 mg/kg HSYA; **(F)** OVA+112.5 mg/kg HSYA; **(G)** OVA+3 mg/kg DXM.

The effect of HSYA on lung mucus secretion was assessed by PAS staining (Figure 6). Mucus was stained with amaranth to distinguish it from other tissues. There was little mucus secretion in the interstitial lung region in the normal control (Figure 6A) and HSYA blank groups (Figure 6B). Mucus secretion was increased in the OVA group (Figure 6C), and this increase was not significantly reduced in the OVA+50 mg/kg HSYA group (Figure 6D). However, OVA-induced mucus secretion in the interstitial lungs was slightly attenuated by 112.5 mg/kg HSYA (Figure 6F) and by DXM (Figure 6G).

### Effect of HSYA on MAPK (ERK, JNK, p38) and IκB Activation

**FIGURE 7:**
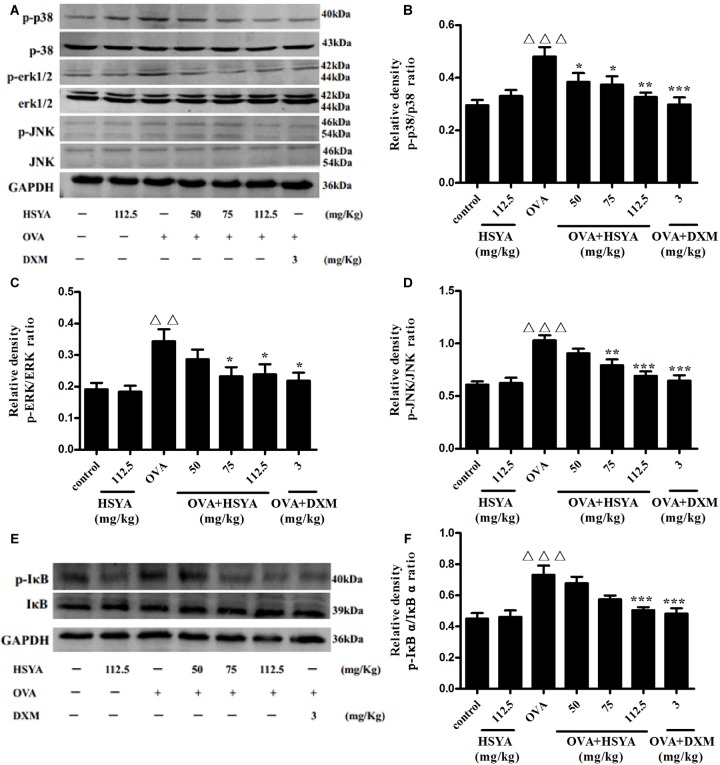
Effects of HSYA on phosphorylated p38 MAPK, ERK MAPK, JNK MAPK, and IκBα in lung tissues by western blot. Protein expression levels were measured by western blotting **(A,E)**. Densitometric analyses of p38 MAPK **(B)**, ERK MAPK **(C)**, JNK MAPK **(D)** and IκBα **(F)**. Data presented as mean ± SD. ^ΔΔ^*p* < 0.01, ^ΔΔΔ^*p* < 0.001 versus normal control group, ^∗^*p* < 0.05, ^∗∗^*p* < 0.01, ^∗∗∗^*p* < 0.001 versus OVA group.

To explore the mechanism of HSYA in treating asthma, we detected the phosphorylation levels of ERK, JNK, p38, and IκBαby western blot. phospho-ERK, phospho-JNK, phospho-p38, and phospho-IκB levels in lung tissue were significantly increased in the OVA group compared with the normal control group (*p* < 0.01) (Figure 7). Levels of phospho-p38 were inhibited by 50 mg/kg HSYA (*p* < 0.05), while phospho-ERK, phospho-JNK, and phospho-p38 were inhibited by 75 mg/kg HSYA (*p* < 0.05), and phosphorylation levels of all the tested factors were inhibited by 112.5 mg/kg HSYA and DXM.

### Effect of HSYA on mRNA Levels of Th1- and Th2-Type Cytokines in Lung Tissue

**FIGURE 8:**
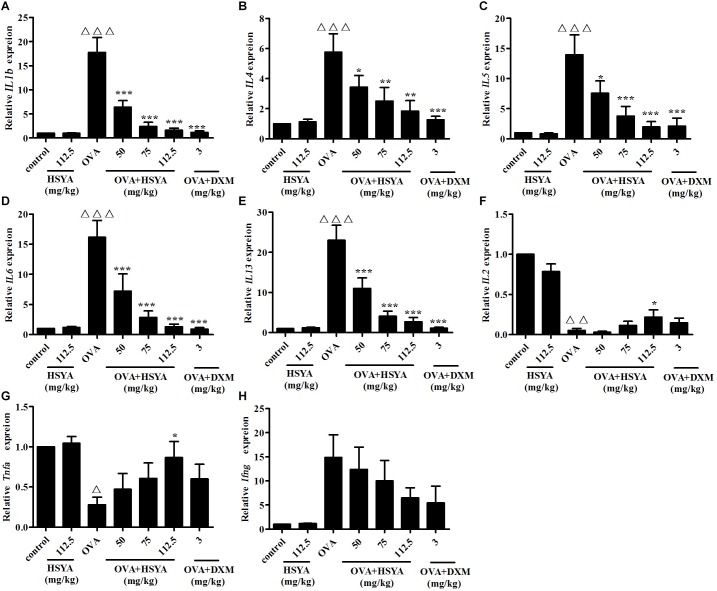
Effects of HSYA on *IL1b* **(A)**, *IL4* **(B)**, *IL5* **(C)**, *IL6* **(D)**, *IL13* **(E)**, *IL2* **(F)**, *Tnfa* **(G)**, and *Ifng* **(H)** mRNA levels in lung tissue in guinea pigs. Data presented as mean ± SD. ^∆^ *p* < 0.05, ^∆∆^ *p* < 0.01, ^∆∆∆^ *p* < 0.001 versus normal control group, ^∗^*p* < 0.05, ^∗∗^*p* < 0.01, ^∗∗∗^*p* < 0.001 versus OVA group.

We detected Th1- and Th2-type cytokines to indicate the balance between the Th1 and Th2 responses. RT-qPCR analysis revealed that the mRNA levels of Th2-type cytokines (*IL13, IL1b, IL6, IL4, IL5*) were significantly increased and the mRNA levels of Th1-type cytokines (*Tnfa* and *IL2*) were significantly decreased in the OVA group compared with the normal control group (*p* < 0.01) (Figure 8). This augmented expression was attenuated by HSYA in a dose-dependent manner and by DXM. The expression levels of *Ifng* mRNA differed significantly among individuals, and although *Ifng* mRNA levels tended to increase, the difference was not significant.

### Effect of HSYA on Plasma IgE, PAF, and Th1- and Th2-Type Cytokine Levels

**FIGURE 9:**
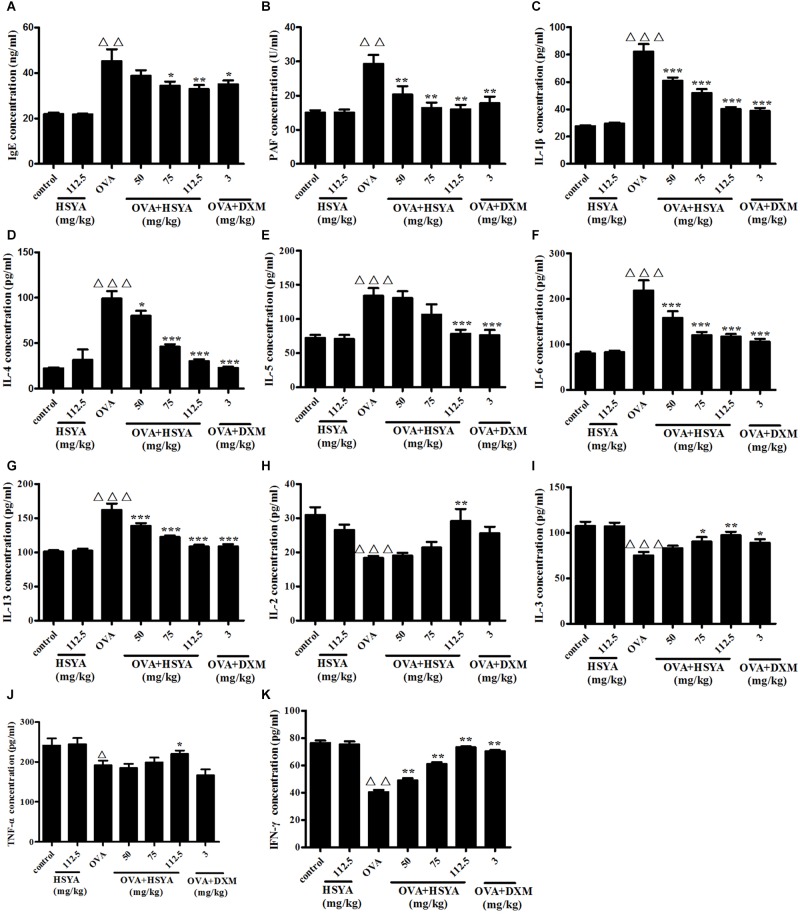
Effects of HSYA on plasma IgE **(A)**, PAF **(B)**, IL-1β **(C)**, IL-4 **(D)**, IL-5 **(E)**, IL-6 **(F)**, IL-13 **(G)**, IL-2 **(H)**, IL-3 **(I)**, TNF-α **(J)**, and IFN-γ **(K)** levels in guinea pigs. Data presented as mean ± SD. ^∆∆^ *p* < 0.01, ^∆∆∆^ *p* < 0.001 versus normal control group, ^∗^*p* < 0.05, ^∗∗^*p* < 0.01, ^∗∗∗^*p* < 0.001 versus OVA group.

Plasma levels of IgE, PAF, and Th2-type cytokines (IL-13, IL-4, IL-6, IL-5, IL-1β) were significantly increased in the OVA group (*p* < 0.01), while levels of Th1-type cytokines (TNF-α, IL-2, IL-3, IFN-γ) were significantly decreased (*p* < 0.05), compared with the control group. HSYA inhibited these increases and decreases, respectively (Figure 9).

## Discussion

Asthma is a complex chronic inflammatory disease of multifactorial etiology, which is characterized by IgE elevation and relatively eosinophilia. Its main pathological features include inflammatory cell infiltration, shedding of epithelial cells, hyperplasia of mucous glands, and proliferation and contraction of airway smooth muscle, resulting in airway narrowing and obstruction. Corticosteroids are commonly used for asthma therapy, which has anti-inflammatory and immunosuppressive effects. Therefore, we used DXM as a positive control drug to evaluate the efficacy of HSYA for the treatment of asthma. In this study, we showed that HSYA could effectively reduce the increase of eosinophil and mucus secretion in lung tissue of asthmatic guinea pigs, and inhibit the synthesis of specific IgE. Besides, we can see in different parts of the study that the effects of high dose HYSA were nearly the same as DXM, and this effects was also demonstrated in our previous researches (Wang Y. et al., 2014; Jin et al., 2016b; Dong et al., 2017). Glucocorticoids are suitable for topical application and long-term systemic use of these drugs can cause serious adverse reactions such as osteoporosis. However, HSYA has the characteristic of multi-targeting, with relatively few side effects, suitable for systemic applications.

Many studies have found that the imbalance of Th1/Th2 response is an important mechanism that causes and exacerbates allergic asthma. IL-13, IL-1 β, IL-6, IL-4, IL-5 are Th2 cell-type factors, mainly involved in mucus secretion, eosinophil production, IgE synthesis, and airway hyperresponsiveness (Guo et al., 2012; Martino et al., 2012). TNF-α, IL-2, IFN-γ, IL-3 are Th1-type factors, whose main function is to inhibit the synthesis of IgE and the differentiation of Th2-type factors (Martino et al., 2012). We showed that the secretion of Th2-type factors (IL-13, IL-1 β, IL-6, IL-4, IL-5) in plasma and lung tissues of guinea pigs in OVA group was significantly increased, while Th1-type cytokines (TNF-α, IL-2, IL-3) were decreased, and this effect was reduced by HSYA. HSYA could thus address the Th1/Th2 imbalance. These results suggest that HSYA may be a potential anti-inflammatory agent for the treatment of asthma.

Platelet activating factor is an inflammatory mediator that plays an important role in the pathogenesis of asthma. PAF has been reported to cause direct bronchial obstruction in animals under experimental conditions, and PAF in plasma of asthmatic patients is significantly higher than that of normal people (Kasperska-Zajac et al., 2008; Gill et al., 2015). Binding of PAF to its receptor induces the activation of multiple second messenger systems, such as phospholipase A2 and phospholipase C, which in turn activate PKC (Takano et al., 1994; Izumi and Shimizu, 1995). PAF can also activate signal pathways such as nuclear factor κB (NF-κB) (Lukashova et al., 2001) and MAPKs (McLaughlin et al., 2006) and enhance the transcriptional activity of activator protein-1 (AP1). In our previous work, we demonstrated that HSYA antagonized PAF-induced changes in these pathways *in vitro* (Guo et al., 2018). In this study, we can see that PAF in plasma of asthmatic group was significantly higher than that of normal group and MAPK signaling pathway was also activated, which aggravates the symptoms of asthma. However, HSYA inhibited these changes *in vivo*. These findings may partially explain the possible mechanism of HSYA in relieving asthma.

Hydroxysafflor yellow A is a water-soluble monomeric constituent isolated from the traditional Chinese medicine *C. tinctorius* L. which has been used to treat blood stasis for 1000s of years. Various researchers have also reported that HSYA inhibited both acute and chronic inflammatory responses (Wang Y. et al., 2014; Dong et al., 2017). These characteristic of HSYA may be beneficial for the treatment of early sensitization and late injury in asthma. Besides, HSYA attenuated pulmonary fibrogenesis induced by BLM in mice (Jin et al., 2016b), and inhibited TGF-β1 signal transduction (Hu et al., 2016). Because TGF-β1 is a key cytokine of the important pathological mechanisms in airway remodeling, thus HSYA may be beneficial for the treatment of airway remodeling in asthma. Safflower yellow injection, which comprises >90% HSYA and HSYA as the main active ingredient, has been widely used in the treatment of coronary heart disease for more than 10 years in China. Traditional Chinese medicine has the characteristic of multi-targeting and can thus intervene in different aspects, with relatively few side effects. Further studies are therefore needed to develop new effective drugs for asthma based on the active ingredients of Chinese medicine.

A weakness of this study was plasma cytokines which studied in experimental animal models. In contrast to tissue, the measurement of cytokines in plasma is difficult to draw straightforward conclusions due to pathology occurs in the tissue. However, we also detected mRNA levels of cytokines in lung tissue and the results are consistent with those of ELISA. Asthma is frequently characterized by airway inflammation rich in eosinophils. Eosinophil degranulation/cytolysis releases the cationic granule proteins, which may contribute to epithelial damage and excessive repair (Acharya and Ackerman, 2014; Persson and Uller, 2014). Furthermore, eosinophils can influence airway inflammation by releasing cytokines (Kelly et al., 2017). In this study, we did not quantify eosinophils, which is major deficiency of the current work.

## Conclusion

The present study revealed that HYSA inhibited OVA-induced asthmatic airway function and immune responses in guinea pigs in a dose-dependent manner. HSYA may alleviate airway function and inflammatory injury in asthmatic by ameliorating the Th1/Th2 cell imbalance, blocking activation of the MAPK signaling pathway, and inhibiting the expression of inflammatory factors such as IgE and PAF. These results suggest that HSYA may play a multifunctional role in controlling the progression of asthma, and is thus worthy of further attention as a potential anti-asthma drug.

## Author Contributions

MZ: experimental implementation and writing the manuscript. MJ: experimental design and manuscript revision. XG and RP: data acquisition and analysis. JG: PAS staining. BZ: analysis of HSYA.

## Conflict of Interest Statement

The authors declare that the research was conducted in the absence of any commercial or financial relationships that could be construed as a potential conflict of interest.
